# The Creation of Articulating Cement Spacer Using Custom-Fabricated Silicone Mold for the Treatment of Periprosthetic Joint Infection: Two Case Reports

**DOI:** 10.7759/cureus.32254

**Published:** 2022-12-06

**Authors:** Jin Chuan Yuen, Ren Yi Kow, Jeffrey Jaya Raj, Chooi Leng Low

**Affiliations:** 1 Department of Orthopedics, Hospital Tengku Ampuan Afzan, Kuantan, MYS; 2 Department of Orthopedics, Traumatology, and Rehabilitation, International Islamic University Malaysia, Kuantan, MYS; 3 Department of Radiology, Sultan Ahmad Shah Medical Centre @International Islamic University Malaysia (IIUM), Kuantan, MYS

**Keywords:** cement spacer techniques, cement spacer, mold, hip and knee replacement, infected knee arthroplasty, infected hip arthroplasty, periprosthetic joint infection, pji

## Abstract

Joint replacement surgeries have been performed to treat joint arthropathies with excellent outcomes. As the number of joint replacement surgeries surges, the incidence of periprosthetic joint infection (PJI) has also increased. Currently, two-stage revision surgery is the gold standard in the treatment of periprosthetic joint infection. Two-stage revision surgery involves joint washout, the removal of the primary implant, the insertion of a cement spacer, and subsequently the reimplantation of prosthesis after the infection has been eliminated. Custom-made articulating cement spacer has been used with success to improve the patient’s ambulatory status and quality of life. Nevertheless, custom-made articulating cement spacer or commercialized cement mold is generally costly. By the modification of previous authors’ techniques, we manage to fabricate reusable silicone molds, which can be used to create articulating cement spacers for both hip and knee joints. We share two case reports to illustrate how these fabricated silicone molds can be a cost-effective technique to create articulating cement spacers to manage periprosthetic joint infection in both hip and knee joints. Surgeons in resource-deprived countries can utilize this technique to create articulating cement spacers with minimal cost, but they need to discuss with their patients and check with the local regulatory board on the feasibility of this technique to create cement spacer that will be used in a patient.

## Introduction

Joint replacement surgeries for the hip and knee are highly reliable and successful in the management of severe osteoarthritis or other arthropathies of the hip and knee joints [1,2]. Total knee arthroplasty (TKA) and total hip arthroplasty (THA) help alleviate patients’ pain, improve their ambulatory status, and improve their quality of life [3-5]. Despite relatively good outcomes in most of the patients with hip and knee joint pathologies, there are several complications associated with these procedures [1-6]. One of the dreaded complications of arthroplasty surgery is periprosthetic joint infection (PJI) [6-8]. The incidence of PJI in the first two years after primary joint replacement ranges from 0.5% to 3%, and revision surgeries show a higher rate of infection, ranging from 2.5% to 4% [1-10].

PJI is associated with high rates of mortality and morbidity, adversely affecting the level of mobility and ambulation of patients, thereby reducing the quality of life [1-10]. Numerous risk factors of PJI have been identified, including advanced age, malnutrition, inflammatory diseases, obesity, diabetes mellitus, and remote infection [1-2]. Despite the best effort to minimize the risk, PJI cannot be completely avoided. The management of PJI can be via single- or two-stage revision surgery [1-2]. Factors precluding single-stage revision are immunocompromised host, concurrent acute local sepsis, soft-tissue or bony compromise not amenable to primary closure, multidrug resistance, and polymicrobial or atypical causative organisms [11]. Furthermore, good results of single-stage revision require a much more aggressive and complete periarticular tissue resection than two-stage, which many surgeons are not accustomed to performing [11]. As there are numerous variables that may adversely affect the outcome of single-stage revision surgeries, two-stage revision is currently considered the gold standard in the management of PJI [1-10].

A temporary cement spacer is imperative in two-stage revision surgery for PJI [9,10]. The temporary cement spacer is inserted after the debridement and removal of the infected primary prosthesis component. On top of its ability to slowly elucidate topical antibiotic, temporary cement spacer also eliminates the dead space left behind by the removal of prosthesis, helping in the eradication of infection and maintaining soft-tissue tension [9,10]. Studies show that static spacers and articulating spacers are comparable in the eradication of infection, but articulating spacers are more superior in providing an improved range of movement of affected joints [9,10]. Custom-made articulating spacer can be made with three dimensional (3D) printing technology, but this can be costly especially in centers with limited resources [12]. Temporary cement spacers have been made with success using silicone molds in the treatment of knee PJI [13]. We share our experience in using this technique to treat PJI of knee and hip joint, respectively, in two patients.

## Case presentation

Case report 1

**Figure 1:**
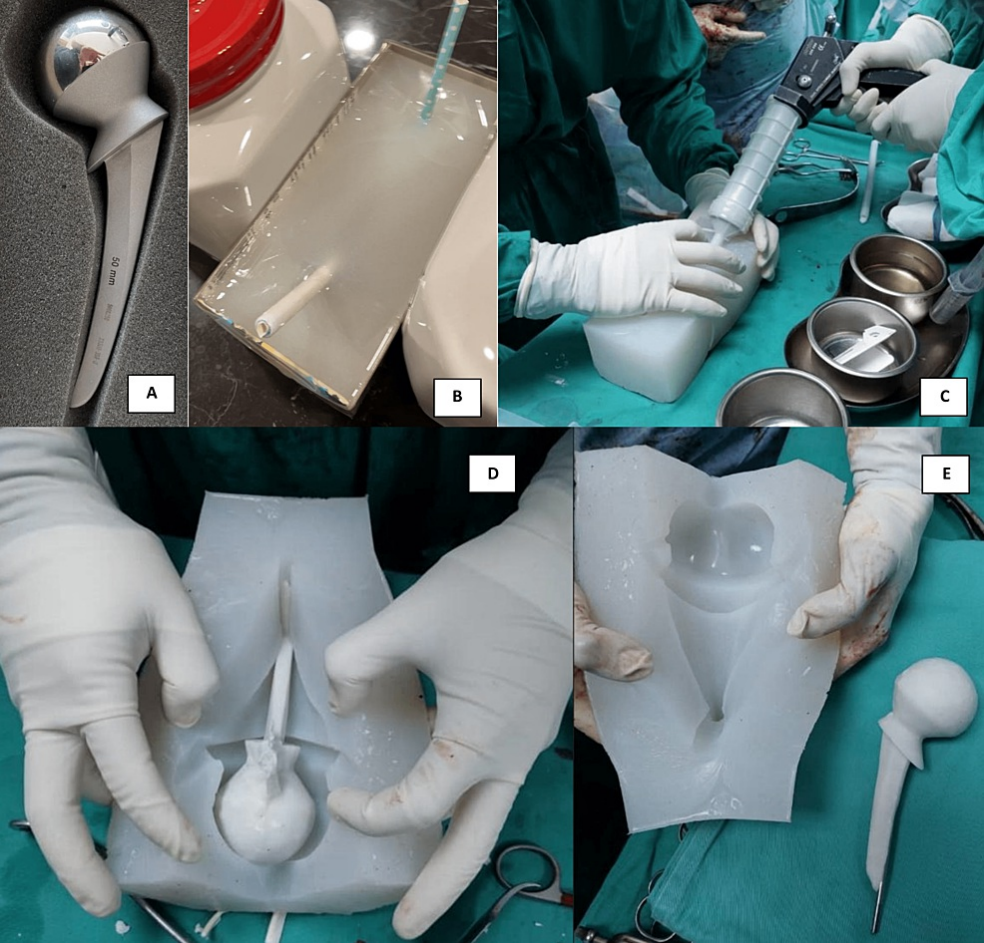
(A) Thompson hemiarthroplasty implant is used to create the silicone mold. (B) Thompson hemiarthroplasty is fully submerged while waiting for the silicone mold to set. (C) Cement is injected into the silicone mold during the waiting phase. (D) The cement spacer is removed after the hardening phase. (E) The silicone mold can be reused after cleaning and sterilization.

Mr. A, a 30-year-old male, was involved in a motor vehicle accident whereby he sustained closed fractures of his left acetabulum and left tibia. He underwent open reduction and reconstructive plating of the left acetabulum and left interlocking nail insertion of the left tibia without any sequelae. Two years later, he presented with left hip pain, and he was diagnosed to have avascular necrosis of the left femoral head. Left total hip replacement (THR) was then performed. Unfortunately, he presented four months after the surgery with periprosthetic joint infection where there is a sinus at the surgical site based on the Musculoskeletal Infection Society (MSIS) criteria. Initially, he underwent wound debridement, sinus excision, and arthrotomy washout. Nevertheless, the attempt to retain the THR implant was futile due to persistent infection. Hence, a two-stage arthroplasty was planned in which an antibiotic-loaded cement spacer was inserted after the removal of the THR implant. Pre-operatively, a Thompson hemiarthroplasty implant (teaching material) was used to create a silicon mold (Figure 1). Intra-operatively, the cement was injected into the silicone mold with a rush rod to reinforce the stability of the cement spacer.

**Figure 2:**
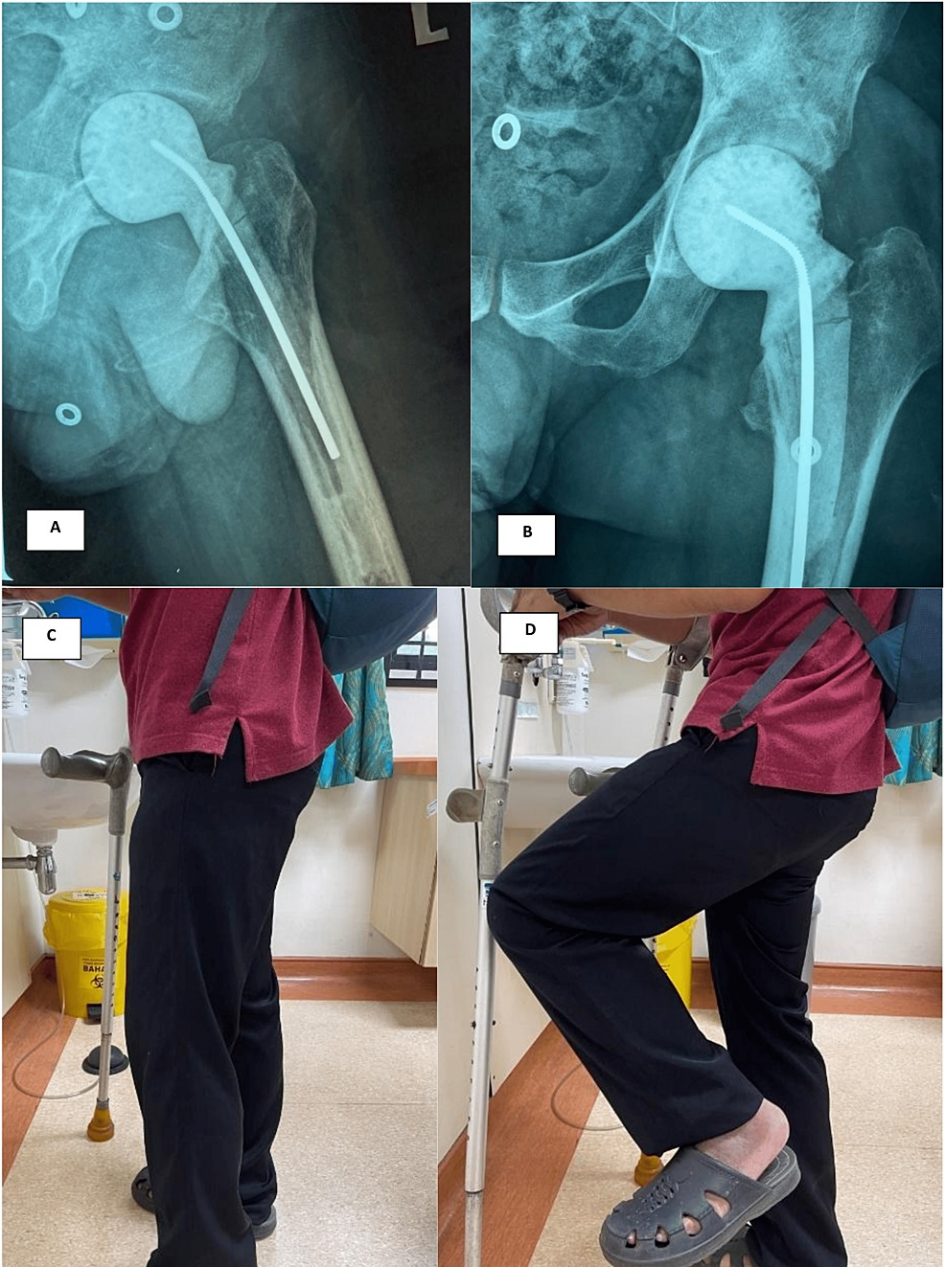
(A) Post-operative plain radiograph in lateral view. (B) Post-operative plain radiograph in AP view. (C) The patient was able to weight-bear with aid. (D) Hip flexion is up to 70 degrees. AP: anteroposterior

Post-operatively, the patient was able to ambulate with partial weight-bearing and hip flexion range of movement measuring 0-70 degrees (Figure 2). Eventually, his infective biomarkers settled down, and he was planned for the revision of total hip replacement later.

Case report 2

**Figure 3:**
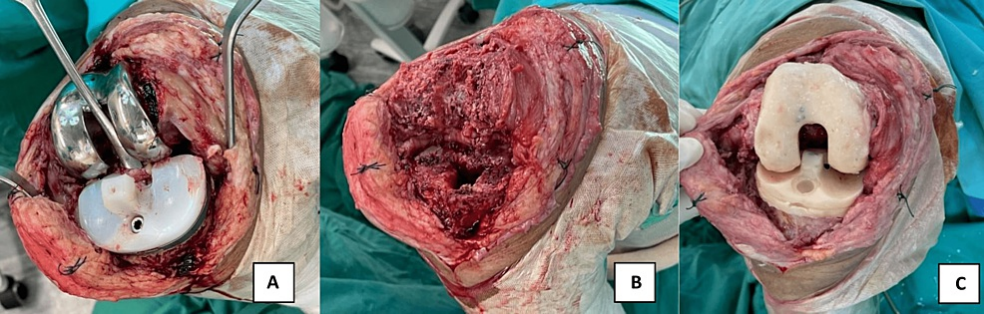
(A) The knee with periprosthetic joint infection is opened. (B) All the infected components are removed. (C) The cement spacer is inserted after thorough debridement and washout of the knee.

Mr. B, a 70-year-old male, underwent right total knee replacement (TKR) for knee osteoarthritis. He was well for three years before he presented with one-month history of right knee pain and swelling. It was not associated with trauma or prior infection. Clinically, his right knee was swollen and warm, and all his infective biomarkers (total white blood cell count, erythrocyte sedimentation rate, and C-reactive protein) were all raised. There was 100 mL of frank pus aspirated from his right knee. The patient underwent debridement, removal of implant, arthrotomy washout, and cement spacer insertion for periprosthetic joint infection of the right knee (Figure 3).

**Figure 4:**
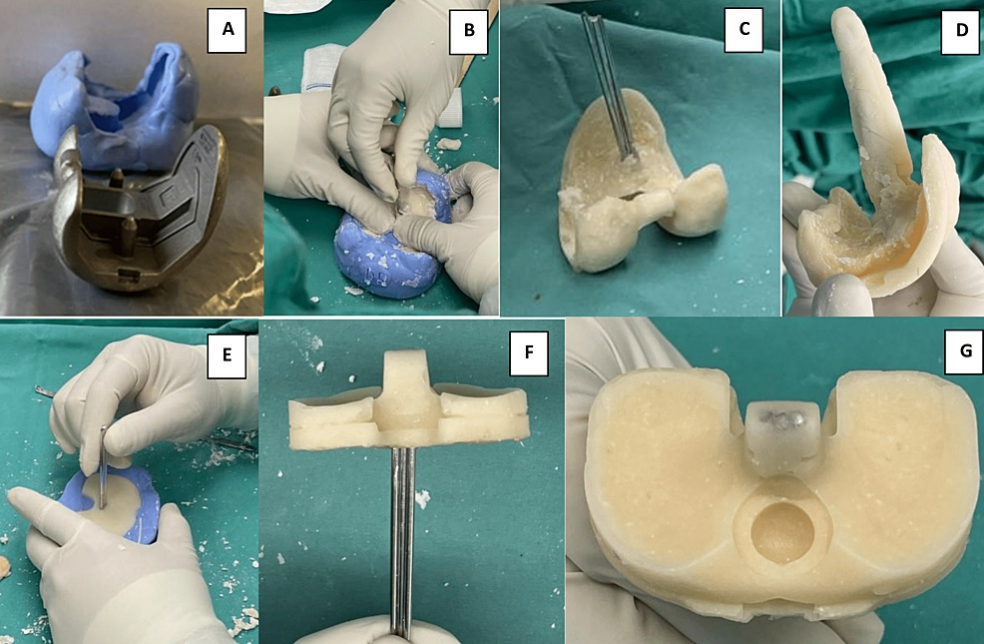
(A) Total knee replacement components are used to create the silicone mold. (B) Cement is injected into the silicone mold, and multiple Kirschner wires are inserted to increase the stability of the spacer. (C) The cement spacer is removed from the silicone mold after the hardening phase. (D) Kirschner wires are covered with cement to increase the surface area of antibiotic elucidation. (E) Similar technique is used to create the tibial part of the cement spacer. (F) The cement spacer is removed after the hardening phase. (G) The cement spacer is inspected prior to implantation.

Pre-operatively, a silicone mold was created using a TKR implant of similar size (Figure 4). Additional silicone molds were also prepared using one-size-up and one-size-down implants. Intra-operatively, cement mixed with vancomycin powder was poured into the silicone putty mold. Three 2.0 mm Kirschner wires were used for additional stability of the stem for both femur and tibial parts of the cement spacer. Post-operatively, the patient was able to weight-bear with an ambulatory aid.

## Discussion

Initially introduced in 1983 by Insall et al., the two-stage revision for PJI involved the removal of all prosthesis components at the septic joint, followed by a course of six-week parental antibiotic therapy and finally replantation with a new prosthesis [1]. In the early 2000s, articulating and static spacers had been introduced to accelerate the eradication of infection [1]. Since then, two-stage revision remains the gold standard for the management of PJI with a success rate of 90%-100% [1-10]. Normally, the antibiotic-loaded cement spacer is inserted for six weeks prior to the second-stage revision [1,2]. Nevertheless, during the COVID-19 pandemic, some patients with PJI had their second-stage revision delayed due to the postponement of elective surgeries in many countries [2]. Hence, it is imperative to have a good-quality cement spacer that allows ambulation while waiting for the second-stage revision.

An antibiotic-loaded cement spacer helps in the eradication of infection through increased local tissue antibiotic concentration [12-14]. Besides that, a well-molded cement spacer can also maintain surrounding soft-tissue tension and prevent further bony damage [12-14]. Custom-made articulating cement spacers further provide additional benefits as they allow patients to be ambulatory while waiting for second-stage revision [12-14]. Nonetheless, commercialized custom-made articulating cement spacers can be cost-prohibitive for centers with limited resources. By using the technique described by Su et al., we have created an articulating cement spacer with silicone mold for a patient with PJI of the knee [13]. Via this technique, silicone molds of TKR prostheses in different sizes are prepared pre-operatively and sterilized for intra-operative usage. We prepare both one-size-up and one-size-down molds in addition to the mold of the exact size of the primary TKR prosthesis, anticipating potential size mismatch due to excessive bone loss.

By using a similar technique, we have successfully created a cement spacer for a patient with PJI of the hip after THR. Like the TKR cement spacer molds, besides the measured head size, we prepare as well one-size-up and one-size-down silicone molds of Thompson hemiarthroplasty heads. In order to increase the stability of the cement spacer, a rush rod is used as a pile reinforcement mechanism within the cement spacer, as described by Kent et al. [14]. In contrast to the technique used by Kent et al. whereby a commercially available one-time-use cement spacer mold was used, our self-made silicone mold has the advantage of reusability after proper cleaning and sterilization, further reducing the cost of creating the cement spacer [14].

An ideal cement spacer should be inexpensive and easily made, permit the elucidation of antibiotics, allow pain-free articulation of the affected joint, and be tough enough to withstand forces during weight-bearing. Via this silicone mold technique, we can create customized cement spacers for both hip and knee joints. This is crucial in the era of the COVID-19 pandemic as the second-stage revision can be postponed for an uncertain period of time [2]. Furthermore, this technique can be used in patients who are not fit for second-stage surgeries due to comorbidities [14].

The silicone used here is an industrial-grade two-part silicone (part A and part B). After mixing the two-part silicone, the mixture is poured over the implant until it is fully submerged (Figure 1B). Multiple straws are inserted until they are in contact with the implant, thereby creating a tunnel up to the implant. The silicone is then left to cure completely, normally up to 24 hours, and the implant is then removed. The silicone mold is then cleaned thoroughly and autoclaved to ensure its sterility. Industrial-grade silicone can withstand heat up to 200°C; hence, sterilization with autoclave will not compromise the silicone mold. The sterilization of the void within the silicone mold was guaranteed via the tunnel created initially with the straws.

Limitation

The generalization of this concept is impossible due to the limited sample size and the lack of control. Future studies can be conducted to compare the effectiveness of this technique with the commercially available molds for cement spacers. Surgeons in resource-deprived countries can utilize this technique to create articulating cement spacers with minimal cost, but they need to discuss with their patients and check with the local regulatory board on the feasibility of this technique to create cement spacer that will be used in a patient.

## Conclusions

Two-stage revision is the mainstay of the management of periprosthetic joint infection of the knee and hip. Articulating cement spacer is crucial for the elimination of infection and to improve the ambulatory status of the patient with PJI. This silicone mold technique can be an inexpensive and effective method to create an articulating cement spacer for both hip and knee joints.
